# Multiscale modeling with GENESIS 3, using the G-shell and Python

**DOI:** 10.1186/1471-2202-13-S1-P176

**Published:** 2012-07-16

**Authors:** Armando L Rodriguez, Hugo Cornelis, David Beeman, James M Bower

**Affiliations:** 1 Barshop Institute , University of Texas Health Science Center, San Antonio, TX 78229, USA; 2 Department of Neurophysiology, Catholic University of Leuven, Leuven, 3000, Belgium; 3 Department of Electrical, Computer, and Energy Engineering, University of Colorado, Boulder, CO 80309, USA

## 

The CBI architecture [1] being used as the basis of GENESIS 3 (G-3) allows a single model-container to be used to describe a model spanning many levels of scale. This feature allows a user to transparently run multi-scale simulations. As will be described in further detail during the workshop “Multi-Scale Modeling in Computational Neuroscience II: Challenges and Opportunities", the CBI architecture contains a communication component to upscale and downscale numerical variables when moving across different levels of scale. These new capabilities and advances in G-3 usability also allow interfacing with many Python graphical tools (e.g. wxPython, matplotlib), potential web interfaces (e.g. Django), and other independent modules (e.g. Chemesis-3) for use in simulations that cover multiple levels of scale. Progress in developing Python interfaces to G-3 [2], combined with recent implementation of network and biochemical modeling capabilities in G-3 have allowed us to construct a new series of self-guided hands-on modeling tutorials. These are being introduced at the Introduction to Genesis 3 Workshop held in Luebeck, Germany 30 April – 5 May 2012 (https://www.gradschool.uni-luebeck.de/index.php?id=366).

This poster provides an introduction to these new modeling capabilities, and to the new instructional material. Additions to the existing G-3 tutorials on use of the G-shell cover network creation commands and the use of the Chemesis-3 module. The rewritten version of the tutorial "Creating large networks with GENESIS" demonstrates the use of Python scripting to create cortical network models in G-3. The tutorial "Adding a GUI to G-3 simulations" shows users how to leverage the Python programming interface to construct visual tools.

As an example, Figure 1 illustrates the use of the new G-3 Netview visualization application to display and replay an animation of the spreading excitation in the RSnet2 simulation that is the basis of the GENESIS network modeling tutorial.

**Figure 1:**
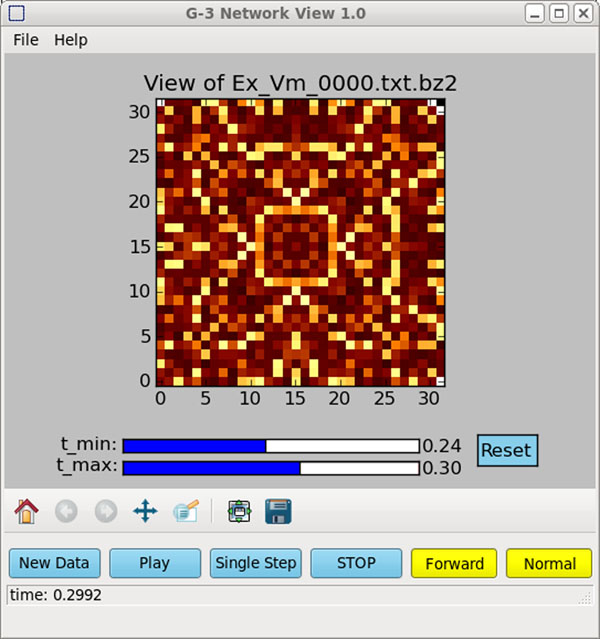
A short injection pulse applied to a 32 x 32 cell network of coupled excitatory cells starts a wave of excitation that can be replayed and examined in detail during any time window, using the G-3 Network Viewer.

## References

[B1] Cornelis H Coop AD Bower JM A Federated Design for a Neurobiological Simulation Engine: The CBI Federated Software Architecture PLoS ONE 2005 7 1 e28956 doi:10.1371/journal.pone.0028956 22242154 10.1371/journal.pone.0028956 PMC3252298

[B2] Cornelis H Rodriguez AL Coop AD Bower JM Python as a Federation Tool for GENESIS 3.0 PLoS ONE 2005 7 1 e29018 doi:10.1371/journal.pone.0029018 22276101 10.1371/journal.pone.0029018 PMC3262781

